# Navigating Research Across Long-Term Care Settings: Strategies for Partnership, Recruitment, and Data Collection

**DOI:** 10.1093/geroni/igaf122.985

**Published:** 2025-12-31

**Authors:** Jasmine Altizer, Abraham Brody

## Abstract

Long-term care settings have long been known as spaces where recruitment of participants and engagement in research including implementation of interventions, surveys, and interviews are challenging. In the same vein, research in these settings is necessary to improve the care of older adults and those who care for them. This symposium will examine innovative methods for conducting research across long-term care settings amid challenges. Key topics include building partnerships with long-term care communities and agencies, adapting research approaches for hard-to-reach participants, and optimizing participant recruitment and data collection that minimizes burden on staff and residents and maintains research rigor and integrity. The session will offer practical insights from researchers working across the long-term care continuum --- in nursing homes, adult day centers, assisted living, home health agencies, and hospice. Dan David will present an innovative strategy to support resident recruitment and facility partnership in Assisted Living through the Facility-Embedded Nurse Researcher model. Tina Sadarangani will share successful approaches to recruitment, intervention design, and implementation of technology in adult day centers. Andreina Jimenez will present strategies to improve research engagement of Black, Latino, and White nursing home residents living with dementia, family care partners, and staff. Chenjuan Ma will present key strategies for navigating challenges related to recruitment, data collection, and stakeholder engagement among providers and aides in home health care. Ab Brody will serve as the discussant for the panel integrating the insights shared across the presentations and adding in approaches to research engagement in Hospice settings. Research in Quality of Care Interest Group Sponsored Symposium

